# Comparison of two negative pressure ureteral access sheaths combined with day-case flexible ureteroscopy for renal stones randomized trial

**DOI:** 10.1038/s41598-024-80934-w

**Published:** 2024-11-23

**Authors:** Qinghong Ma, Guoqiang Chen, Guanghai Li, Deheng Cui

**Affiliations:** Department of Urology, The Second Hospital of Longyan, Longyan, 364000 Fujian China

**Keywords:** Intelligent intrarenal pressure control platforms, Flexible ureteral access sheath, Flexible ureteroscopic lithotripsy, Negative pressure, Urology, Ureter

## Abstract

To compare the safety and effectiveness of the combination of intelligent intrarenal pressure control platforms and flexible end ureteral access sheath combined with flexible ureteroscopic lithotripsy (FURL) for the treatment of renal stones less than 2 cm with day case mode. From November 2023 to July 2024, a prospective, randomized, double-blind, parallel-controlled study was conducted to recruit 60 patients with upper urinary tract calculi measuring ≤ 2 cm in longest diameter. A total of 60 eligible patients were consecutively enrolled. Based on a pre-generated random number table and allocation scheme using SPSS 27.0, patients were assigned to either the intelligent pressure control group (IFURL, *n* = 30) or the head bending group (BFURL, *n* = 30). Blinding measures were implemented for patients, data collectors, statisticians, and analysts, with the allocation results disclosed to the surgeons prior to the start of the procedure in the operating room. The operation duration for IFURL and BFURL was 52.50 (48.00, 60.00) vs. 46.00 (36.00, 56.25), respectively (*p* = 0.047). Stone free rate (SFR) on the first postoperative day was 73.33% vs. 93.33% for IFURL and BFURL, respectively (*p* = 0.038). While, SFR was no significant difference between the two groups after two months(90.00% vs. 96.67%, *p* = 0.301). One patient in IFURL was readmitted after discharge due to fever 2 days post-discharge, and improved after 2 days of anti-infection treatment. One patient in BFURL was readmitted due to lumbar and abdominal pain from urine extravasation, which improved after 3 days of anti-infection treatment. SFR of one-month post-surgery for renal calculi ≤ 2 cm treated with intelligent pressure control and flexible UAS combined with FURL in day surgery mode was similar, with low infection-related complications and rehospitalization rates, showing no statistical difference. However, the overall hospitalization costs for the BFURL was lower than IFURL.

**Supplementary Information:**

The online version contains supplementary material available at 10.1038/s41598-024-80934-w.

## Introduction

Urolithiasis is one of the most common diseases of the urinary system. The prevalence of urolithiasis varies widely across different regions, ranging from 1–13%^1^. The incidence of urolithiasis in Chinese adults is as high as 5.8%^2^. Recent evidence suggests that the prevalence of urolithiasis is increasing globally, leading to a growing economic burden associated with the diagnosis, treatment, and complications of the disease^1^. Percutaneous nephrolithotomy (PCNL) and flexible ureteroscopic lithotripsy (FURL) are the main methods for treating upper urinary tract calculi. To enhance bed utilization and reduce treatment costs, some medical centers have implemented day surgery for FURL, which can reduce hospitalization time, lower costs, and improve patient satisfaction^3^.

Due to the short duration of hospitalization for patients undergoing day surgery, there is still a high rate of readmission after discharge, mainly due to fever, sepsis, and renal colic^3^. In short, how to further reduce the risk of infection and improve stone free rate(SFR) has always been a research hotspot. Although FURL has the advantages of passing through natural orifices, minimal trauma, and rapid recovery, some disadvantages limit its further promotion, such as poor water circulation in traditional ureteral access sheaths(TUAS), blurred vision, and dependence on self-removal of stones after surgery^4^. Most notably, perioperative infection complications such as fever, sepsis, and septic shock had been reported to occur in up to 37%^5^.

In recent years, the use of ureteral access sheaths with negative pressure suction(NUAS), in conjunction with disposable flexible ureteroscopes, for lithotripsy has rapidly expanded in China. This technique consistently maintains low intrapelvic pressure(IPP) and swiftly removes stones, offering significant advantages over traditional FURL^6,7^. Our center had conducted a retrospective study to assess the safety and efficacy of two negative pressure devices, in combination with FURL, for treating upper urinary tract stones^8^. The study found that both devices had low infection-related complications and demonstrated a statistically significant difference in immediate stone removal rates. However, no significant difference was observed one-month post-surgery. In this study, we designed a prospective, randomized, controlled trial to evaluate the safety and effectiveness of two different adjustable negative pressure suction devices, in conjunction with FURL, for treating upper urinary tract stones during daytime surgery. We also aim to share our insights into daytime surgical practices.

## Methods

### Patients

 From November 2023 to July 2024, a prospective, randomized, double-blind, parallel-controlled study was conducted to recruit 60 patients with upper urinary tract calculi measuring ≤ 2 cm in longest diameter. All procedures and participants in the human-involved research strictly adhered to the ethical standards set by the Institutional Research Committee and the 1964 Helsinki Declaration and its subsequent amendments. The research protocol was approved by the ethics committee of the Second Hospital of Longyan City, Fujian Province (LYEY2023LSK-025). The study had been registered in the Chinese clinical trial registry with registration number: ChiCTR2400088490, 20/08/2024. All patients provided informed consent after being informed of the benefits and risks associated with each procedure.

Inclusion criteria: ① Aged 18–60 years; ② Calculi located in the kidney and/or upper ureter, with a single stone diameter of ≤ 2 cm, or a cumulative maximum diameter of multiple stones of ≤ 2 cm; ③ ASA score I/II; ④ All patients voluntarily agreed to participate in the study and provided informed consent. Exclusion criteria: ① Uncontrolled urinary tract infection; ② Patients with pyonephrosis identified during surgery; ③ Patients with anatomical abnormalities (ectopic kidney, horseshoe kidney, duplex kidney), ureteral stenosis, urethral stricture, or urinary diversion; ④ Severe hydronephrosis; ⑤ Renal function in the decompensated phase (serum creatinine > 178umol/L); ⑥ Severe systemic hemorrhagic disease; ⑦ Patients undergoing simultaneous bilateral surgery; ⑧ Severe hip deformity resulting in a difficult posture. Assuming at least 30 patients in each group, with α = 0.05 and test efficiency of 1 − β = 80%, a total of 60 patients completed the study, with no patients lost to follow-up^8^.Based on a pre-generated random number table and allocation scheme using SPSS 27.0, patients were assigned to either the intelligent pressure control group (IFURL, *n* = 30) or the head bending group (BFURL, *n* = 30). Blinding measures were implemented for patients, data collectors, statisticians, and analysts, with the allocation results disclosed to the surgeons prior to the start of the procedure in the operating room.

### Preoperative preparation

Prior to surgery, all patients in both groups underwent biochemical tests, coagulation studies, urinalysis, urine culture, a kidney-ureter-bladder X-ray, and a non-enhanced CT scan of the urinary system. Data were collected for both groups, including age, gender, BMI, comorbidities, characteristics of the stones, prior surgical history, pre-existing catheters, urine culture results, perioperative complications, and hospitalization costs. Patients with positive preoperative urine cultures were treated with antibiotics sensitive to the drug sensitivity results until the culture became negative. Patients with negative preoperative urine cultures received a single dose of antibiotics for prophylactic infection. Surgery in each group was performed by the same experienced surgeon specializing in stone removal.

### Discharge criteria and follow-up plan

According to the postanesthesia discharge score (PADS), whether the patient can leave the hospital or not is determined. The total score was 10 points, and those who score ≥ 9 points could leave the hospital. Postoperative unobstructed urination, absence of gross hematuria, low back pain, abdominal pain, fever, chills, independent ambulation, no dysphagia or cough, no dizziness or headache, no nausea or vomiting, clear and pertinent responses, and confirmation of normal double-J tube position on the abdominal X-ray.

Patients’ conditions including hematuria, low back pain, chills, fever, and others were monitored at 24 h, 48 h, 72 h, 1 week, 2 weeks, and 4 weeks post-surgery by telephone or wechat. On the first day postoperation, SFR was evaluated by abdominal X-ray. At the 2 month postoperation, renal NCCT was performed to confirm the final SFR. DJ stent were put out in 2 week postoperatively.

### Observation indicators

The primary endpoint was the SFR at 2-month post-operation, with no residual stones or fragments smaller than 2 mm considered as successful clearance.Secondary endpoints included operation duration and perioperative complications such as fever, sepsis, septic shock, and urinary leakage.

### Surgical procedure

#### IFURL group

**Fig. 1:**
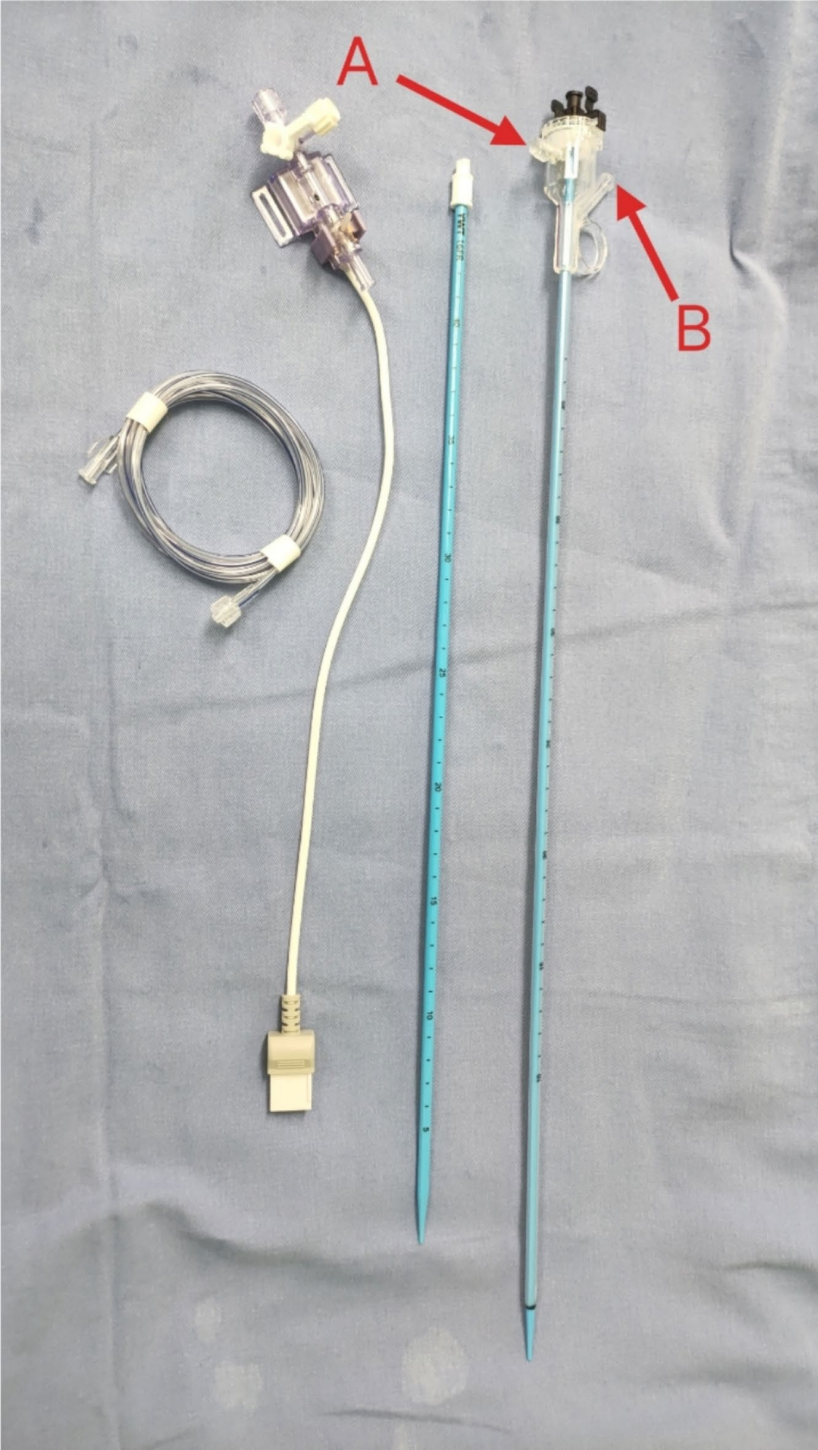
Ureteral access sheaths and pressure conduction equipment of intelligent pressure control platform (Red **A** is the pressure monitoring connection port, red **B** is the negative pressure suction connection port).

All patients received general anesthesia and were in the oblique supine position. The urethra and ureter were examined using rigid ureteroscopy, with the length of the UAS determined by the depth of the ureteroscopy. The pressure sensor was connected to the irrigation pipeline, and the pressure was calibrated. Intrapelvic pressure alarm was set at 30mmHg, with an irrigation flow rate of 50-150 ml/min. Tip of the 12/14F UAS (Fig. 1) was positioned at the ureteropelvic junction. A disposable flexible ureteroscope (Zebra F8.6) was utilized alongside a 200 μm holmium laser fiber for lithotripsy, with energy set at 0.6–0.8 J and frequency at 20–30 Hz. Irrigation fluid was used to create a vortex in the renal calyces to maximize the extraction of stone fragments. Postoperatively, a 7 F double J tube was left in place, with a urinary catheter left at the end of the procedure.

#### BFURL group

**Fig. 2:**
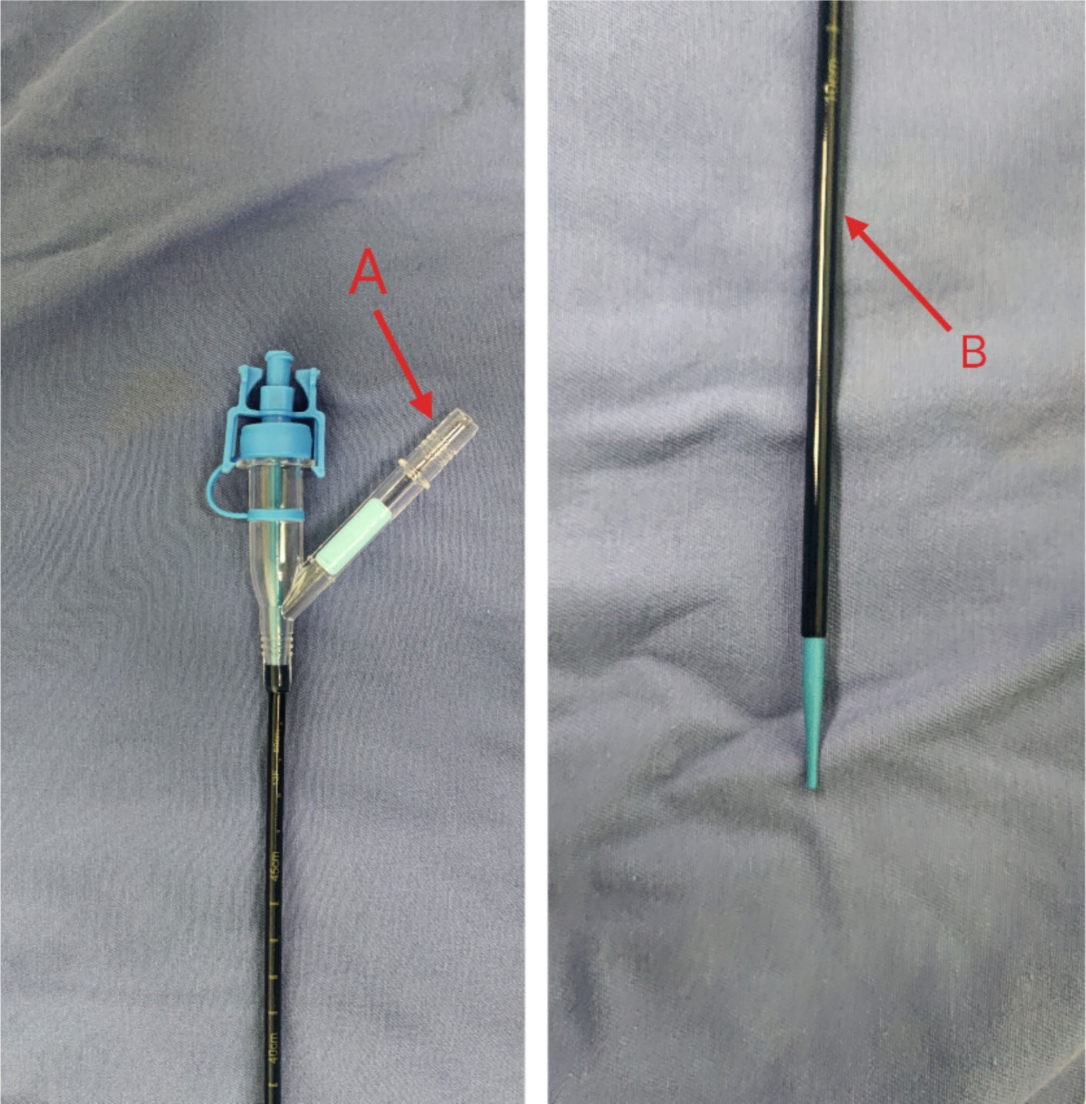
End flexible ureteral access sheaths (Red **A** is the negative pressure suction connection port, red **B** is bendable end).

All patients received general anesthesia and were in the oblique supine position. A rigid ureteroscope was used to inspect the urethra and ureter. A 12/14F disposable flexible UAS (Fig. 2) was inserted into the renal pelvis via the guide wire. The sheath’s tail was connected to central negative pressure suction platform, set at 0.02–0.04 MPa. A disposable flexible ureteroscope (Zebra F8.6) was employed, with irrigation flow set at 50–150 ml/min. During the procedure, 200 μm holmium laser fiber was utilized, with energy at 0.6–0.8 J and frequency at 20–30 Hz. The flexible tip of the UAS was used to access each renal calyx for the extraction of stone fragments. Post-lithotripsy, a 7 F double J catheter was left, with a urinary catheter remaining at the end of the surgery.

### Statistical methods

Measurement data were initially tested for normality and equality of variances. If the data followed a normal distribution and had equal variance, two independent sample t-tests were performed, with results expressed as mean ± standard deviation. If the data did not follow a normal distribution or had unequal variance, the Mann-Whitney U test was used, and results were expressed as the median (upper quartile to lower quartile). Count data were expressed as cases (%), the Mann-Whitney U test was used for ordinal count data, and the χ^2^ test was used for non-ordinal count data. *P* < 0.05 was considered statistically significant. All statistical analyses were conducted using the commercially available SPSS 27.0 software.

## Results

All patients completed the surgery and followed the scheduled follow-up plan.No significant differences were observed in general characteristics between the two groups, including age, gender, BMI, comorbidities, stone lateral location, stone position, stone length, urine culture, and hydronephrosis (Table 1). The operation duration for IFURL and BFURL was 52.50 (48.00, 60.00) vs. 46.00 (36.00, 56.25), respectively (*p* = 0.047).SFR on the first postoperative day was 73.33% vs. 93.33% for IFURL and BFURL, respectively (*p* = 0.038). While, SFR was no significant difference between the two groups after two months(90.00% vs. 96.67%, *p* = 0.301).In IFURL, there was 1 case of fever, 1 case of sepsis, and 1 case of urine extravasation. There were two cases of mild injury to the ureteral mucosa in each group, with no cases of ureteral perforation.

**Table 1:** General information of the two groups.

Variable	IFURL group	BFURL group	*P* value
Age (year)	49.50[42.75,55.25]	47.50[37.25,54.00]	0.501
Gender, n(%)			0.432
Male	16(53.30)	14(46.70)	
Female	19(63.30)	11(36.70)	
BMI (kg/m^2^)	23.45 ± 3.03	24.29 ± 2.88	0.165
Urine culture n (%)			0.389
Positive	2(6.67)	4(13.33)	
Negative	28(92.33)	26(86.67)	
Comorbidities, n(%)			0.718
None	26(86.67)	25(83.33)	
Diabetes	4(13.33)	5(16.67)	
Stone site, n(%)			1.000
Right	15(50.00)	15(50.00)	
Left	15(50.00)	15(50.00)	
Stone location, n(%)			0.951
Renal	10(33.33)	10(33.33)	
Ureter	10(33.33)	9(30.00)	
Renal and ureter	10(33.33)	11(36.67)	
Stone diameter(mm)	13[11.00,15.00]	12.5[10.00,15.00]	0.489
Stone CT density (HU)			0.598
≤ 1000	19(63.33)	17(56.67)	
> 1000	11(36.67)	13(43.33)	
Hydronephrosis, n(%)			0.504
None	12(40.00)	12(40.00)	
≤ 1 cm	12(40.00)	11(36.67)	
1–2 cm	5(16.67)	3(10.00)	
> 2 cm	1(3.33)	4(13.33)	
ASA score, n(%)			0.688
I	27(90.00)	26(86.67)	
II	3(10.00)	4(13.33)	
Pre-DJ stent, n(%)	3(10.00)	4(13.33)	0.688

BMI, body mass index; HU, hounsfield units.

In BFURL, there was 1 case of fever, no sepsis, and 1 case of urine extravasation. Neither group experienced perirenal hematoma, renal artery embolism, septic shock, or patient deaths.One patient in IFURL was readmitted after discharge due to fever 2 days post-discharge, and improved after 2 days of anti-infection treatment. One patient in BFURL was readmitted due to lumbar and abdominal pain from urine extravasation, which improved after 3 days of anti-infection treatment (Table 2). The summary of IPP for the IFURL group were shown in Table 3.

**Table 2:** Perioperative complications and postoperative effect evaluation of two groups.SFR, stone-free rate.

Variable	IFURL group	BFRUL group	*P*
Mean operative time (min)	52.50[48.00,60.00]	46.00[36.00,56.25]	0.047*
Fever, n(%)	1(3.33)	1(3.33)	1.000
Spesis, n(%)	1(3.33)	0	0.313
Urinary leakage, n(%)	1(3.33)	1(3.33)	1.000
Total SFR at first day, (%)	73.33	93.33	0.038*
Total SFR at one month, (%)	90.00	96.67	0.301
Readmission, n(%)	1(3.33)	1(3.33)	1.000
Hospitalization expenses, (RMB)	16,548 ± 1977	14,847 ± 1903	0.001*
Ureter dilation, n(%)	4(13.33)	3(10)	0.688

*p < 0.05, statistically significant difference.

**Table 3:** Summary of intraoperative intrapelvic pressure(IPP) date in intelligent pressure control group.

Variable	Mean	Rang	Standard deviation
Mean IPP, mmHg	9.2	5–15	2.51
Maximal IPP, mmHg	24.6	17–35	0.52

## Discussion

Day surgery significantly reduces hospital stays, enhances bed utilization and resource efficiency, lowers nosocomial infections, and alleviates both patient financial burdens and staff workloads. Suhonen et al. found that day surgery notably improves patients’ quality of life during recovery and prevents nosocomial infections^9^. Our center has preliminarily explored the use of negative pressure technology combined with FURL for treating renal calculi smaller than 2 cm in the day surgery setting, and we share our findings in this article. Currently, negative pressure suction devices are classified into intelligent and non-intelligent pressure control types based on their control methods. The intelligent type, requiring a dedicated control platform costing 300,000 to 500,000 RMB, is more expensive. The non-intelligent type, widely used in China, requires only NUAS with a connectable negative pressure interface, linked to the operating room’s central negative pressure system, significantly reducing costs. However, non-intelligent devices have notable drawbacks: they cannot display precise pressure values and must rely on the surgeon’s experience, posing safety challenges.

Urinary tract infections are characterized by rapid progression and poor prognosis, with sepsis or septic shock significantly increasing mortality rates^10^. Traditional UAS irrigation solution is challenging to remove and can be blocked by stone debris, leading to elevated renal pelvis pressure^11^. When the pressure in the renal pelvis exceeds 30mmHg, the risk of infection rises significantly^12^. By contrast, UAS with negative pressure suction capabilities offer advantages in maintaining low renal pelvis pressure, reducing toxin absorption, and lowering the incidence of infection^7^. Currently, the flexible-end UAS with non-intelligent, pressure-controlled, as well as the intelligent pressure-control platform UAS, are widely used and have demonstrated safety and effectiveness^8^. Fever occurred in one patient in each group (3.33%), with one case of sepsis in IFURL and none in BFURL. Still, this difference was not statistically significant (*P* = 0.313), both being lower than the 5% incidence rate associated with traditional FURL. Maintaining low renal pelvic pressure during surgery was crucial in reducing the incidence of infection. The two devices require different techniques: ①IFURL maintains low renal pelvis pressure while generating a pressure differential through rapid liquid circulation and creating a vortex through directional irrigation fluid adjustment, effectively removing stone fragments. ②In BFURL group, real-time pressure monitoring was not feasible, and maintaining a negative pressure within the renal pelvis was based on the following criteria.Firstly, the renal pelvis and calyces exhibited slight invagination instead of being full. Secondly, if the field of vision suddenly became turbid or fluid flow noticeably decreased, immediate suspension of the procedure was required to check for equipment blockages. In the event of improper use and blockage, the intelligent pressure control device automatically discontinues fluid infusion, unlike the BFURL group without a feedback mechanism, where continuous infusion could lead to a sharp increase in renal pelvis pressure, an outcome we aim to avoid.

SFR on the first-day post-operation in BFURL was higher than in IFURL, with a statistically significant difference. However, at one-month post-operation, no significant difference was observed in the SFR. The observed differences may be attributed to the following factors: ① In BFURL, the tip of the UAS was positioned as close to the stone as possible, facilitating efficient and straightforward stone extraction. ② In IFURL, the stones were extracted by the vortex generated by the irrigation fluid, which increased the difficulty of stone removal. It was important to note that both techniques involve withdrawing the scope along with water jets to flush out the stones, rather than relying on increased negative pressure suction to extract the stones. The fragments after negative pressure technique FURL were basically removed, which reduces the risk of failed self-stone removal, because even fragments smaller than 2 mm carry a risk of failed removal, and the process of eliminating small fragments may also cause pain, hematuria, and fever^13,14^. In addition, the UAS end of BFURL can directly access most renal calyces, reducing the need for stone baskets. In contrast, the UAS end of IFURL located at the UPJ, necessitates more frequent use of stone baskets, thereby increasing the overall hospitalization costs for patients.

Day surgery offers superior medical services to patients, significantly reducing the duration of treatment. Fewer complications lower the likelihood of unplanned readmissions, enhancing surgical safety and facilitating technology adoption. Based on our experience, not all patients are candidates for this technology; effective communication and adherence to post-discharge follow-up plans are required. Common postoperative discomforts include pain and hematuria. Pain levels must be assessed upon discharge, and analgesics should be prepared as needed. Hematuria is related to both the procedure and postoperative activities and diet, should be managed by avoiding strenuous activities and cautious use of anticoagulants or blood-activating foods. Before discharge, clear follow-up arrangements, including the use of mobile phones and chat software, must be established according to the predetermined follow-up schedule and topics.

Although our study is a prospective, randomized controlled trial, it is a single-center study with a small sample size, which may result in a lack of sufficient confidence in the statistical analysis of the data. Patients with advanced age and high ASA grade (ASA > II) were excluded from the study. Whether these patients reach the same conclusion requires further investigation. A future multi-center, large-sample randomized controlled trial is planned.

## Conclusion

SFR of one-month post-surgery for renal calculi ≤ 2 cm treated with intelligent pressure control and flexible UAS combined with FURL in day surgery mode was similar, with low infection-related complications and rehospitalization rates, showing no statistical difference. However, the overall hospitalization costs for the BFURL was lower than IFURL.

## Electronic supplementary material

Below is the link to the electronic supplementary material.

Supplementary Material 1

## Data Availability

Data is provided within the manuscript or supplementary information files.
